# Detection, Diagnosis and Treatment of Acute Ischemic Stroke: Current and Future Perspectives

**DOI:** 10.3389/fmedt.2022.748949

**Published:** 2022-06-24

**Authors:** Smita Patil, Rosanna Rossi, Duaa Jabrah, Karen Doyle

**Affiliations:** ^1^CÚRAM, SFI Research Centre for Medical Devices, National University of Ireland Galway, Galway, Ireland; ^2^Department of Physiology, National University of Ireland Galway, Galway, Ireland

**Keywords:** stroke, thrombectomy, neuroradiology, devices and intervention, CT, MRI, portable devices

## Abstract

Stroke is one of the leading causes of disability worldwide. Early diagnosis and treatment of stroke are important for better clinical outcome. Rapid and accurate diagnosis of stroke subtypes is critical. This review discusses the advantages and disadvantages of the current diagnostic and assessment techniques used in clinical practice, particularly for diagnosing acute ischemic stroke. Alternative techniques for rapid detection of stroke utilizing blood based biomarkers and novel portable devices employing imaging methods such as volumetric impedance phase-shift spectroscopy, microwave tomography and Doppler ultrasound are also discussed. Current therapeutic approaches for treating acute ischemic stroke using thrombolytic drugs and endovascular thrombectomy are discussed, with a focus on devices and approaches recently developed to treat large cranial vessel occlusions.

## Introduction

Stroke is the second largest cause of mortality worldwide, only surpassed by ischemic heart disease (1). According to the World Stroke Organization, over 13.7 million stroke attacks are reported each year, of those cases 60% are under the age of 70 (1, 2). The lifetime risk of having a stroke in people who are 25 years and older is 24.9% (2). More than 2.7 million people die from ischaemic stroke attacks each year (2). The damage to the brain can be inflicted through blockage (ischaemic stroke) or rupture of a cerebral artery (hemorrhagic stroke). Acute ischaemic stroke (AIS) is the most common, accounting for approximately 85% of cases (2). Thromboembolism associated with large artery atherosclerosis or cardiac diseases such as atrial fibrillation are the most common etiologies (1).

Occlusions of internal carotid artery (ICA), M1 or M2 segment of the middle cerebral artery (MCA), or vertebro basilar arteries called large vessel occlusions (LVO) represent 11%-29% of AIS (3, 4). Brain hypoperfusion caused by a LVO commonly results in irreversible death of brain tissue, due to a lack of blood supply to an area, leading to the core infarction. The core is surrounded by the penumbra, brain tissue that is hypoperfused, but can be salvaged by prompt restoration of blood flow (1). In this regard, endovascular treatment (EVT) has been successfully used for recanalization in LVO (5–7).

The most common stroke symptoms people present with are facial numbness and weakness, visual impairment, weakness of the upper or lower limbs on one side of the body, impaired balance, nausea, abrupt severe headache of an unknown cause and speech impairment (1). These traditional symptoms are reported to present equally in men and women, but women are more likely to present with non-traditional stroke symptoms like light headedness and loss of consciousness (8). Fast, accurate diagnosis of stroke is vital for selection of appropriate acute stroke treatment, such as intra venous tissue plasminogen activator (IV tPA) or endovascular mechanical thrombectomy treatment.

For rapid diagnosis and triage, various field triage stroke scales have been developed. These scores have been validated for clinical use and allow rapid diagnosis of patients suspected to be suffering from acute ischemic stroke (9). Based on the clinical examination utilizing stroke scales, the patient is transferred to thrombectomy centers, if required. Such transfers are significantly costly (10), and a false positive diagnosis adds further burden on the health system. An assessment of the capability of 13 clinical scores for identifying LVOs in over 1,000 patients found high false negative and false positive rates (11). The highest accuracy observed was 79%, when NIHSS score ≥11 was utilized for diagnosis (11). Meta-analysis showed that no scale was capable of identifying LVO with high sensitivity and specificity (12). High false-positive rates of 50–65% have been observed (12). Therefore, it is clear that field triage stroke scales are insufficient for accurate diagnosis alone.

## Current State-of-the-Art Techniques for Stroke Assessment

The current gold-standard approach to assessment of stroke requires brain and neurovascular imaging, in addition to the clinical assessment of stroke severity using National Institute of Health Stroke Scale (NIHSS). Advantages and disadvantages of all imaging techniques used for assessment of stroke are summarized in Table 1.

**Table 1:** Imaging techniques used for assessment of AIS.

**Imaging technique**		**Use in stroke**	**Benefits (✓) and drawbacks (×)**
Non contrast computed tomography (NCCT)		Diagnosis of major stroke. Identify a contraindication to treatment, like hemorrhage or large infarct. Identify stroke mimic such as brain tumor	✓ Fast and widely available **×** Insensitive to the diagnosis of minor stroke due to low spatial resolution (13) **×** Moderate interrater reliability, even between experts (14)
Computed tomography angiography (CTA)		Detection of large vessel occlusion (LVO)	✓ Detects LVO with almost 100% sensitivity **×** Constrast agent is required (typically iodine)
	Single-phase CT angiography (sCTA)	Allows the assessment of the global colateral circulation (15)	✓ Rapid evaluation of the presence of intracranial LVO **×** Single image; no temporal resolution
	Multiphase CTA (mCTA)	Provides time-resolved images of the cerebral vasculature with 3 cerebral image acquisitions	✓ Can be acquired in 30 seconds, thus lesser motion artifacts ✓ Good interrater reliability ✓ Excellent predictor of clinical outcome (16) **×** Higher radiation dose compared to sCTA but lower radiation dose than CTP **×** Interpretation requires high degree of expertise
CT perfusion (CTP) or perfusion computed tomography (PCT)		Time-resolved images of blood flow Display format is perfusion maps, including CBF, CBV, and MTT. Used to identify and quantify volume of infarcted core and penumbra	✓ Easier to interpret than CTA ✓ Easy availability, rapid acquisition **×** Long image-acquisition times (continuous scanning for 45–90 seconds) **×** Susceptible to motion artefacts **×** More radiation exposure compared to CTA as additional contrast dose required **×** Complex acquisition and heterogeneous postprocessing algorithms (17)
Digital subtraction angiography (DSA)		Old technology. Classical evaluation of circulation status	**×** Time consuming invasive technique, only justified in the case of mechanical thrombectomy **×** Bilateral carotid and vertebral injections are required
MRI		Most sensitive technique for AIS detection. Used in imaging diagnosis of minor stroke and typically during follow-up (18)	✓ Excelllent spatial resolution ✓ Can detect brain ischemia in transient ischemic attack or minor ischemic stroke ✓ Can be conducted without the application of contrast agents (DWI, SWI, ASL) but for early diagnostic purposes contrast agents are used **×** Time-consuming, fastest protocol takes 6 min (19) **×** Susceptible to motion artefacts **×** May not be available 24 h in many centers
	Diffusion MRI or diffusion-weighted imaging (DWI)	Used to obtain the perfusion fraction. Used to accurately quantify volume of infarcted core	✓ High (73–92%) sensitivity 3h and ~100% sensitivity 6h after onset (20) **×** ADC values can be affected by flow apart from diffusion (21)
	Susceptibility weighted imaging (SWI)	Used to evaluate cerebral microbleeds	**×** Artifacts can be produced from patient motion
MR angiography (MRA)		Used in patients with a contraindication to contrast or a non-diagnostic CTA (22)	**×** MRA is not needed if CTA is performed
	Non-contrast enhanced MRA time-of-flight (TOF)	Measures and illustrates the flow of blood inside vessel structures in 2D and 3D (23)	**×** Poor depiction of some areas or thrombus shine through might lead to misdiagnosis of occlusion (24)
	Non-contrast enhanced MRA. Phase-contrast	Non-contrast-enhanced MRA with a high rate of background suppression. Used for better visualization of cerebral blood flow	✓ Good spatial resolution
	Contrast enhanced-MRA		✓ Shorter acquisition time than non contrast MRA ✓ less susceptible to patient motion **×** Uses gadolinium-based contrast agent **×** Requires intravenous access and contrast administration **×** Decreased spatial resolution compared to TOF (20)
MR Perfusion	Arterial spin labeling (ASL)	Used to quantify volume of infarcted core (using either DWI or ASL). Used for estimating brain perfusion to core and penumbra	✓ Prefered over CTP when available to avoid radiation exposure ✓ Non-contrast-enhanced technique **×** Sensitive to susceptibility artifacts, motion artifacts

*ICA, internal carotid artery; MCA, middle cerebral artery; EVT, endovascular therapy; CBF, cerebral blood flow; CBV, cerebral blood volume; MTT, mean transit time; CC, colateral circulation; ADC, apparent diffusion coefficient; IVIM, intravoxel incoherent motion imaging*.

Non-contrast computed tomography (NCCT) of the head is fast, widely available, and cost effective. It is used as the primary imaging in patients with suspected AIS to eliminate acute hemorrhage (25). NCCT images are widely used to assess the site and extent of AIS using the Alberta Stroke Program Early CT Score (ASPECTS) (26). Interpretation of NCCT images by an expert can diagnose major stroke but is markedly insensitive in diagnosing minor stroke (18). NCCT also has low sensitivity (<20%) in the first 3 h and 57–71% sensitivity 24 h after stroke onset for detecting AIS (20).

CT angiography (CTA) and CT perfusion (CTP) are routinely used for diagnosis and selecting patients for endovascular therapy (EVT) (27). Multimodal CT protocols including CT, CTA and CT perfusion are now available in many hospitals, and are used to identify the occluding vessel and assess collateral flow in AIS. The major drawback of this method is the time required to carry the sequential imaging, but advancement of automated software is facilitating in time reduction. While imaging time is increased with CTP/CTA, one study showed that overall treatment time was not increased with CTA and CTP in comparison to NCCT only, probably due to quick assessment and better anatomic data prior to EVT (28).

Single phase CT angiography (sCTA) is useful for the rapid evaluation of LVO, and is also useful for evaluation of collateral circulation. Good collaterals on sCTA correlate with reduced ischemic core growth, although no correlation between collateral status and clinical outcome was observed (29). NCCT is relatively insensitive in detecting early ischemic change in the vertebrobasilar region, so additional CTA is essential for diagnosing vertebrobasilar ischemia (3, 20). CTA immediately followed by NCCT is recommended for all presentations of acute stroke syndromes (18). Many EVT trials have used CT and CTA for selecting patients and utilized ASPECTS for estimating the extension of established infarction. It can be problematic to compare sCTA data from different studies due to inconsistency in the timing of the contrast injection and image acquisition (16). Furthermore, diverse collateral circulation scoring methods for sCTA have been suggested but there is no accepted standard evaluation method (30).

Multiphase CTA (mCTA) gives three time-resolved gray-scale images of the cerebral vasculature, with 8 and 16 s delays. Thus, the reader needs to link these images together for visualization and interpretation, which necessitates a high degree of expertise. mCTA provides consistent evaluation of pial artery filling in AIS imaging and has good interrater reliability as well as predicts clinical outcome with remarkable accuracy (16). mCTA is better for detecting distal vessel occlusion than sCTA alone (31). Color-coded mCTA summation maps may simplify AIS pathology assessment, including improved evaluation of collateral status, carotid pseudo-occlusions, distal occlusions, intracranial stenosis, and giving an indication of permeability of thrombus (17).

CT perfusion (CTP) provides comparable diagnosis and prognosis capabilities to mCTA and is often preferred as the color maps used are easier to interpret (17). CTP also uses time-resolved images of parenchymal blood flow and a single color-coded cerebral map of projected blood flow to display a probable tissue fate using sophisticated acquisition and post-processing tools. CTP gives a similar estimate of mismatch compared to MR perfusion-diffusion (32). However, differences in CT scanners can result in variation in performance and CT scanners with smaller detector size cannot give whole-brain coverage.

CTP assesses blood flow at the capillary tissue level using data from repeated cerebral CT scans. Perfusion parameters utilized in identifying core and penumbra comprise cerebral blood flow (CBF), cerebral blood volume (CBV), mean transit time (MTT), time to peak (TTP), and time to maximum (Tmax). Although there is no clear consensus in the literature on the exact parameters or thresholds to be used for characterizing core and penumbra, brain tissue with severely reduced CBV or CBF predicts infarcted core, and regions with prolongation of the MTT or its derivatives, the TTP or Tmax, determine the penumbra (4, 33).

Digital subtraction angiography (DSA) is an old technology that requires invasive action and is not widely used today. In DSA pre-contrast images taken on fluoroscope are subtracted from the contrast images for better visualization of vasculature. DSA can also be used to determine occlusion location and evaluate collateral circulation, most commonly during EVT. DSA is more invasive than CTA and the interrater reliability of CTA-determined occlusion type has been reported to be better than DSA-determined occlusion type, so CTA is more widely used (34).

Magnetic resonance imaging (MRI) has greater sensitivity than any other imaging techniques for AIS detection. However, it is not as widely available as other imaging modalities and it is mostly utilized as follow-up imaging (18). Prompt screening of patients for MRI compatibility can be difficult in AIS patients with severe neurologic symptoms. Use of MRI in AIS patients is problematic due to longer imaging times and availability issues, nonetheless, numerous institutions utilize MRI for AIS patients in the hyperacute setting (22). MRI, mCTA, and CTP have all been found to be excellent techniques to detect core and penumbra in recent clinical trials (7, 35, 36).

Diffusion MRI or diffusion-weighted imaging (DWI) is the gold standard for the imaging diagnosis of AIS, detecting it as early as 30 min after the beginning of symptoms. In DWI, diffusion of protons in the tissue is shown and tissues with delayed or restricted proton movement with a low apparent diffusion coefficient (ADC) appear bright. By using intravoxel incoherent motion imaging (IVIM), DWI can be employed to obtain the perfusion fraction, which is related to the cerebral blood volume (CBV) (37). While NCCT, CTA and CTP can be utilized for estimating or deducing the size of core and penumbra, DWI gives more sensitive and specific measurement of acutely infarcted brain tissue volume (38). Additionally, DWI has been found to predict outcome in posterior circulation stroke (39). Furthermore, arterial spin-labeled (ASL)-DWI mismatch can identify salvageable tissue (40). However, IVIM perfusion estimates are contentious and susceptible to echo time effects (20).

Susceptibility-weighted imaging (SWI), fluid attenuated inversion recovery (FLAIR) and T2-weighted MRI, can be utilized for accurate identification of hemorrhage and other stroke mimics (41, 42). MR images obtained with FLAIR demonstrate variable signal intensity up to 6 h from onset and high signal intensity after 6 h (43). A 6-min multimodal MRI protocol utilizing a combination of echo-planar imaging (EPI) and parallel acquisition technique on a 3T MR scanner has shown good diagnostic sensitivity for AIS patients (19). In SWI, phase difference due to magnetic susceptibility between deoxygenated and oxygenated blood is exploited to illustrate cerebral vasculature (37). SWI or T2-weighted MRI can be employed to evaluate cerebral microbleeds, which some have proposed should be considered during tPA treatment decisions (44, 45).

Magnetic resonance angiography (MRA) can be used as an alternative tool to CTA to assess brain perfusion, although it is not as widely used as CTA. Both sCTA and MRA permit quick detection of large vessel occlusion (LVO) (46). Overall, the performance and reading of sCTA and MRA imaging data is technically not more challenging than perfusion imaging and is effective for AIS treatment decisions. MRA is used in patients with a contraindication to contrast or when a non-diagnostic CTA is obtained during evaluation of cerebral vasculature (22). Contrast-enhanced MRA has been shown to exhibit greater accuracy than non-contrast enhanced MRA for identifying intracranial arterial occlusion (47).

The 2018 Guidelines for Management of Acute Ischemic Stroke from the American Heart Association/ American Stroke Association now recommends that CTP, diffusion-weighted imaging (DWI)- MRI, and/or MRI perfusion (MRP) be included as part of a standard imaging evaluation for patients within 6–24 h of symptom onset (48). Diagnosis of stroke mimics requires use of MRI or CTP.

Although there is some agreement on the parameters that define favorable and unfavorable perfusion profiles, the selection criteria for intervention continues to be ambiguous. CTP and MR perfusion characteristics denote complex metabolic changes during AIS in a simplistic manner. Thus understanding of how these characteristics are associated with patient outcome is required (49). For example, a tissue with defined penumbra characteristics on CTP, might not be salvageable even after successful recanalization if it is already at a stage where cell death and infarction are unavoidable (25).

## Recent Medical Technological Advances for Detection of Stroke and Stroke Subtypes

CT and MRI, while widely employed for diagnosing AIS, are not available in every healthcare facility. There is considerable ongoing research and development for novel, practical, portable, economical and complementary instruments to the currently used diagnostic techniques in acute care units. A variety of novel portable devices are being developed for diagnosis of stroke with good sensitivity and specificity. Diagnostic devices in development employ alternative imaging methods such as volumetric impedance phase-shift spectroscopy, microwave tomography and Doppler ultrasound and are in various stages of prototype development and testing (9, 50, 51).

**Figure 1:**
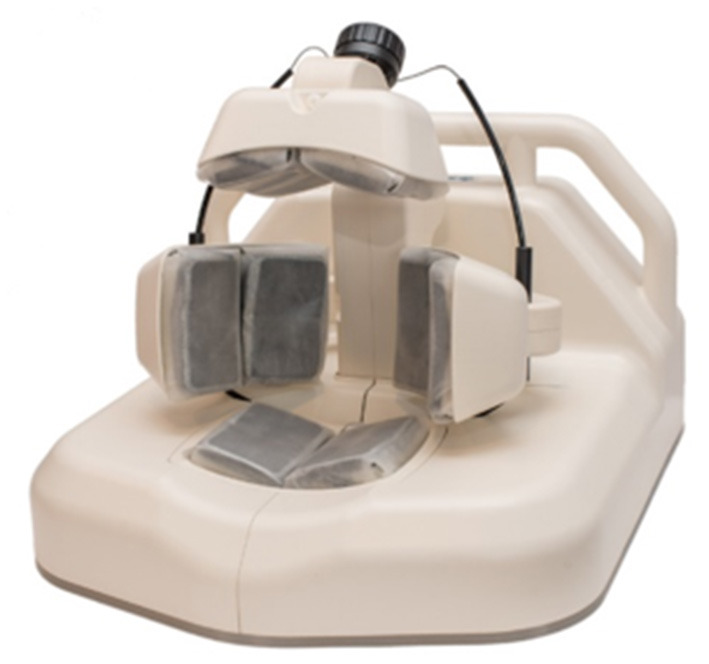
The Strokefinder MD100 device (Medfield Diagnostics AB).

Strokefinder MD100 developed by Medfield Diagnostics AB (Gothenburg, Sweden) is a microwave based device that fits on a stretcher and can identify intracranial hemorrhage with high sensitivity (100%) and specificity (75%) (50, 52). Microwave based technology has been explored for more than three decades in the biomedical field and it is safe. While it has not been used much for diagnostic purposes, it can easily penetrate skull compared to ultrasound and impedance. This technology uses low power non-ionizing microwave radiation along with mathematical algorithms for signal processing. The Strokefinder device was tested on stoke patients as well as healthy volunteers. 65% of AIS patients could be differentiated from intracranial hemorrhage using the microwave device (52). The measurement takes around 45 sec in Strokefinder MD100 (50). The device consists of eight antennas mounted in four pairs as shown in Figure 1.

Transcranial Doppler ultrasound based technology measures cerebral blood flow velocity. The Lucid™ Robotic System using transcranial Doppler ultrasound (Neural Analytics, California, USA) has been reported to differentiate patients with LVOs with high sensitivity (100%) and specificity (86%) from healthy controls (51). It also showed 91% sensitivity and 85% specificity, when diagnosing LVOs in patients suspected to have stroke (51). The Lucid™ system has been FDA approved in USA and CE marked in the European Union.

**Figure 2:**
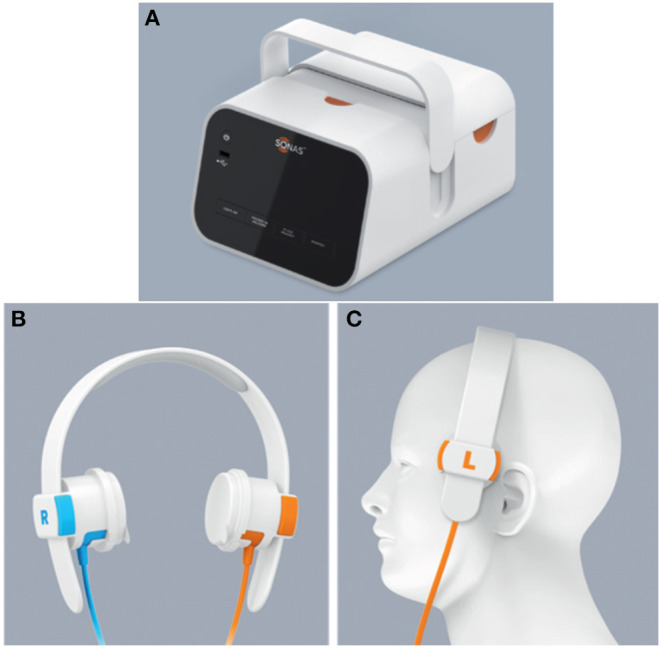
The SONAS® device (BURL Concepts, California, USA), **(A)** Signal generator, **(B)** headphones and **(C)** headphone placement over patient's head.

The SONAS® (BURL Concepts, California, USA), also utilizes transcranial ultrasound to detect LVOs. SONAS® helps in unilateral and bilateral assessment of cerebral blood perfusion (Figure 2). However, this device uses commercially available contrast agents (e.g., Lumason®/SonoVue®) that are injected intravenously to detect the LVOs. These contrast agents are microbubbles containing sulfur hexafluoride that are used for enhancing imaging of internal body structures during ultrasound scans. The SONAS® device includes a headset with ultrasound transducers positioned on each side of head. Each side has two individual transducers packed together, one element to transmit and the other to receive signal. SONAS® transmits ultrasound at a low, sub MHz frequency in an alternating fashion right vs. left. SONAS® measures brain perfusion in both hemispheres using microbubbles as acoustic tracers. Similar to MRI- or CT-Perfusion, the contrast agent is administered as a bolus injection. The change in microbubble related acoustic signal strength is monitored over time and displayed as bolus kinetic curves. This portable device is currently undergoing clinical trials (9).

**Figure 3:**
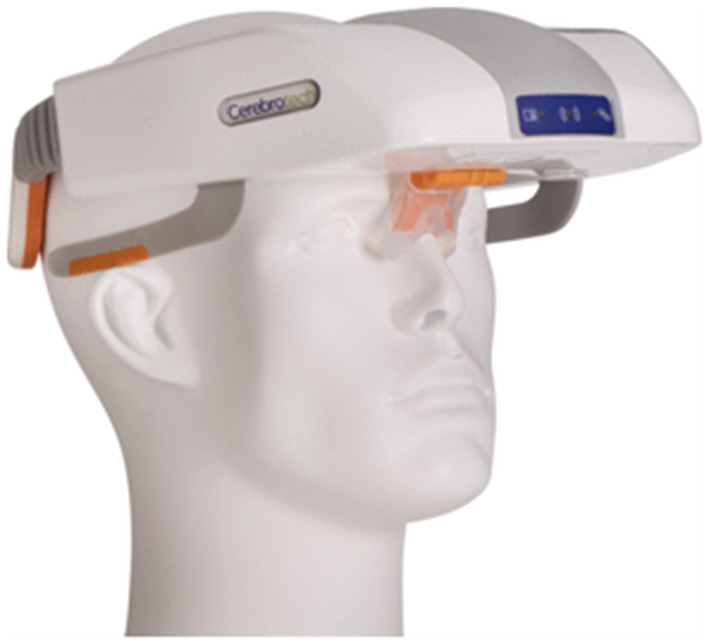
The volumetric impedance phase shift spectroscopy based Visor™ device (Cerebrotech).

A volumetric impedance phase shift spectroscopy (VIPS^TM^) based Visor® device by Cerebrotech (California, USA), showed high sensitivity (93%) and specificity (92%) and was able to differentiate severe stroke from minor strokes caused by LVO in a study with 248 subjects (51). Visor® is a non-invasive device that is placed on the patient's head to detect changes in bioimpedance (Figure 3). VIPS technology can detect bioimpedance signature across a tissue. The device works by transmitting a spectrum of low energy radio waves from each side at the back of the head. These waves are then received by the receiver placed at the front of the head. When the radio waves pass through the tissue, they are modified differently based on tissue type and properties of fluid present in the tissue, thus giving unique signatures for different brain pathologies. VIPS can detect alterations in bioimpedance properties of brain in LVOs due to edema and changes in electrolytes. This Visor® device is approved by FDA for distribution in the USA, and is CE marked for distribution in the European Union (51).

Electrical impedance tomography (EIT) is also an impedance based imaging technique that is portable, safe, and economical (53). EIT creates images of internal electrical impedance of a tissue by using numerous measurements of radiofrequency (KHz to several MHz) currents passed through electrodes present on the surface of the tissue (53). A single measurement is taken by passing current through a pair of electrodes and measuring the resulting voltages with other electrodes. Then multiple measurements are obtained quickly by alternating the electrodes which pass current and measure it. Mathematical models are employed for reconstructing the tomographic images of internal bioimpedance of the tissue (54). EIT was initially developed for imaging the thorax (54), and it is currently employed for monitoring lung ventilation in hospitals (55).

EIT of brain is being studied for its potential to identify the location of epileptic foci (56), and also for its potential in rapid stroke diagnosis, such as identifying stroke type (57), localization and monitoring of intraventricular and intracranial hemorrhage, ischemia and cerebral edema (58–60). It has been shown that there is a difference in impedance of healthy and stroke-affected brain (i.e., ischemic or hemorrhagic brain), in animal models using EIT (61, 62). EIT was successfully used to monitor focal cerebral infarction in a rabbit model (63). A clear difference in impedance measurements in healthy brain, ischemic brain and blood was observed using four terminal electrode measurements for EIT in frequency range of 10 Hz−3 kHz in rats (62). In further investigations, using a rat model of MCA occlusion and 3D EIT with 40 spring-loaded cranial electrodes (64), increased voltage measurements were observed upon MCA occlusion but many artifacts were present in the reconstructed images, thus infarcted cores could not be identified in all cases (64). A further recent study showed that EIT could be used for real time monitoring of local impedance changes in rat brain following MCA occlusion (65). Goren et al. reported the first multi frequency EIT data in human from 23 stroke patients and 10 health volunteers (53), demonstrating the potential of EIT for clinical use in the detection of AIS in future.

A panel of accurate blood biomarkers could be an important additional diagnostic tool (66–73). The characteristics for the ideal stroke blood biomarkers should first of all have high specificity and sensitivity similar to the characteristics of biomarkers observed using imaging tools. This is critical to distinguish stroke from stroke mimics and to differentiate hemorrhagic and ischemic stroke. Ideal biomarkers should be involved in cellular processes that are unique to ischemic stroke, which is very challenging. Levels of proteins that are normally highly expressed in brain, but not in blood are obvious potential candidates.

During stroke, blood brain barrier dysfunction reduces the compartmentation between the fluids of the brain and periphery, thereby allowing movement of brain-derived factors into the blood that would normally be prevented. Necrosis in the infarct core can result in the release of glial structural proteins in to the blood stream at considerably higher than normal levels. Glial proteins such as glial fibrillary acidic protein (GFAP), and S100β, which are both structural astrocytic proteins, are two very promising blood biomarkers that could be indicative of stroke in patients presenting with symptoms of acute neurological dysfunction. Furthermore, evidence suggests that levels of GFAP and S100β could distinguish between AIS and brain hemorrhage. GFAP is considered the best candidate to date for differentiating hemorrhagic and ischemic stroke (71). Several studies performed in different clinical settings showed a delayed GFAP release in patients with ischemic stroke, compared to hemorrhagic stroke, with a maximum concentration reached 2–4 days after ischemic stroke onset (74–77). Although there is less clear consensus in the literature, S100β has shown promising results as a possible biomarker to distinguish AIS from brain hemorrhage (78–80). There is evidence that peripheral S100β level gives an estimation of extent of brain damage generally, not specifically for stroke, as its levels are increased also with other neurological conditions (81). However, in conjunction with acute and sudden neurological symptoms, it is likely that it could be useful diagnostically for stroke. High S100β levels (>0.23 g/L) in blood in ischemic stroke patients are associated with a higher risk of hemorrhagic transformation after thrombolysis treatment (82), which makes this protein of great interest for informing clinical decision-making after stroke, even though further studies are needed.

Finally, several studies and meta-analyses have described other possible biomarkers for stroke diagnosis, including matrix metalloproteinase 9 (MMP-9) (83, 84), Brain natriuretic peptide (BNP) (85), N-methyl-D-aspartate receptor proteins (86) and apolipoproteins (87, 88), which may also help to discriminate between atheroembolic stroke and other main etiologies (89). To achieve a biomarker assay with both high sensitivity and selectivity it may be necessary to use a combination of several biomarkers in a panel (90, 91). However, much more investigation needs to be done to validate these and other possible stroke biomarkers to lead to a viable panel for clinical diagnostic use.

## Therapeutic Approaches to Acute Ischemic Stroke

To date, the only therapeutic approaches approved for treatment of AIS are the administration of a thrombolytic therapy and the mechanical removal of the occlusive clot through endovascular mechanical thrombectomy (MT) (92–95).

Blood clot formation is a physiological response to vessel injury starting with formation of a loose platelet plug, followed by activation of the coagulation cascade that results in the formation of a fibrin mesh that strengthens the clot and allows the injured vessel to be repaired. Clot dissolution then naturally occurs over the next several days as the wounded blood vessel wall heals, through the activation of plasminogen to plasmin, a serine protease that is a powerful fibrinolytic. Tissue plasminogen activator (tPA) is responsible for the cleavage of plasminogen to plasmin, which is slowly released by endothelial cells at the site of injury.

The first record of the use of molecules naturally implicated in the normal clot dissolution to treat acute ischemic stroke occurred in 1958 (96). Three patients were treated with intravenous plasmin over a period of 6 days, with notable symptomatic improvement observed in one patient (96). However, the potential of thrombolytic agents to treat acute ischemic stroke did not advance to the clinic for many years due to concern over the increased risk of hemorrhage. In 1995, a key clinical trial proved tPA to be a beneficial thrombolytic treatment for AIS (97) and the Food and Drug administration subsequently approved it for use to treat AIS. Until recently, alteplase, the recombinant form of tPA (rtPA), was the only therapeutic option for AIS. Alteplase has a very short life of 4–6 min in the body, due to the potent actions of plasminogen activator inhibitor protein (PAI-1) (98); for this reason, it must be administered intravenously. rtPA has to be administered within a tight time-window of 4.5 h from stroke onset to minimize risk of side effects such as hemorrhage (99). rtPA administration minimizes the likelihood of disability 3 months after treatment by 30%. However, successful revascularization of the occluded blood vessel by thrombolytic treatment alone occurs in less than half of cases (99). It seems that histological composition of the clot could be an important factor in predicting whether rtPA administration will be successful or not (100). Clots richer in platelets may be more resistant to thrombolysis (101), while RBC-rich thrombi seem to have higher sensitivity to rtPA (102, 103).

**Figure 4:**
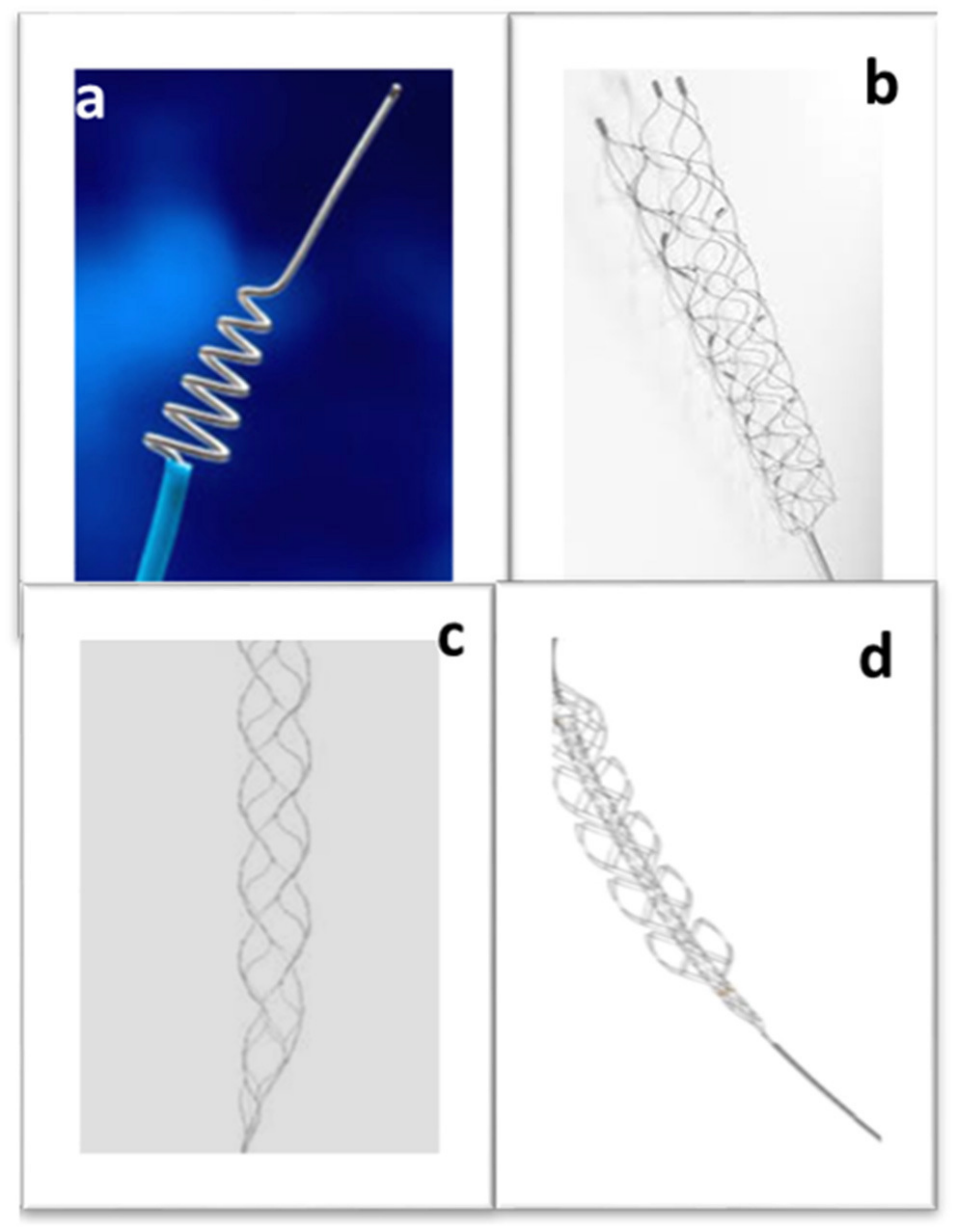
Stent retrievers. The Merci retriever **(a)**, the Solitaire **(b)** and the Trevo device **(c)**. The Embotrap **(d)** with its visible inner stent-like channel (Images were acquired from www.tdg.ucla.edu, www.medtronic.com, www.stryker.com, www.jnjmedicaldevices.com respectively).

Mechanical thrombectomy is an endovascular procedure involving the introduction of a medical device into the vasculature *via* the groin, threading it through the heart to reach the cerebral vessel occluded by the blood clot and mechanically removing it, allowing the restoration of the natural blood flow to the brain. Five clinical trials in 2015 demonstrated the effectiveness of mechanical thrombectomy as an AIS treatment, and it is fast becoming the gold standard. However, initial attempts to mechanically manipulate the clot using microcatheters and percutaneous balloon angioplasty devices highlighted the risk of distal embolization (104). The 2005 MERCI trial, however, gave promising results and the Merci retriever (Figure 4a) became the first FDA approved mechanical thrombectomy device for AIS treatment (105). The Penumbra system was the second thrombectomy device to be approved by the FDA a few years later, showing safe and successful recanalization in AIS patients who were treated within 8 h of experiencing symptoms (106). The Merci retriever and the Penumbra device are the ancestors of the two main approaches currently used in mechanical thrombectomy for clot removal: respectively employing a stent retriever (stentriever) or an aspiration catheter. Aspiration catheter and stentriever can be used alone (107), with a balloon guide catheter to stop blood flow during intervention (108), or in combination (109).

To date, with the advance in endovascular procedures, there is a huge debate about what is the optimal treatment choice for patients with suspected AIS, i.e., whether thrombolysis or immediate mechanical thrombectomy should be prioritized (110). A great advantage of rtPA is that the treatment is readily available at stroke units that do not have a mechanical thrombectomy service or that cannot provide a 24 h service (111).

Although many developments have occurred since the Merci retriever, the general way stentrievers work remains the same. They are minimally invasive, catheter-based devices designed to physically entrap the clot allowing its subsequent removal. A microcatheter is used to deploy the stentriever at the occlusion site. During the thrombectomy procedure, the neurointerventional radiologist guides the microcatheter to cross the clot, the retrievable stent is then unsheathed from the microcatheter and as it deploys, it meshes with the clot. The metallic stent is allowed to interact with the clot for typically 2–4 min and then the stent and microcatheter are retrieved, pulling out the clot. In 2012, the FDA approved the Solitaire device (Figure 4b), which achieved better recanalization outcomes than the Merci device (112). The Trevo device (Figure 4c), was also approved in the same year (113). However, the first clinical trials using the first generation intra-arterial devices yielded negative results (114–116) and almost derailed the future of mechanical thrombectomy to treat stroke. As previously mentioned, it was as recent as 2015 that five randomized clinical trials demonstrated positive results, leading to the acceptance of mechanical thrombectomy as a gold-standard therapeutic approach for AIS (5, 6, 35, 117, 118). Further developments in stentriever device design led to devices such as the Embotrap (Figure 4d), which contains an inner stent like channel that can entrap the clot fragments, which may reduce the incidence of distal embolization (119).

In aspiration thrombectomy, a catheter is advanced through the vasculature to the occluded vessel, placing the distal end of the catheter proximal to the clot. The application of a negative pressure with a syringe or a pump allows the clot to be suctioned into the catheter. In the past, the use of aspiration thrombectomy in AIS treatment was limited by the lack of catheters large enough to provide proper aspiration, yet flexible and atraumatic enough to navigate the tortuous intracranial vasculature. With newer aspiration catheters, however, these obstacles have been overcome and aspiration thrombectomy has been shown to give comparable results to stentriever devices in terms of recanalization outcome (120) with the advantage of being less expensive than stentrievers and enabling a quicker thrombectomy procedural time (121), which is a particularly important consideration.

## Future Perspectives and Conclusion

Diagnosis and assessment of stroke and its subtypes is currently very dependent on the use of gold-standard imaging techniques such as CT and MRI. Availability of these imaging facilities is limited in economically poor regions, small hospitals and clinics. Delays in stroke assessment and treatment leads to a worse outcome for the patient. The development of portable devices and diagnostic biomarker panels to detect stroke and distinguish hemorrhagic and ischemic subtypes could have widespread use if they are found to be useful in clinical decision making. These devices could allow earlier assessment of stroke by first responders responding to a medical emergency in the field. The high cost of CT and MRI scanners and the low level of access to imaging facilities and expertise, particularly in poorer parts of the world, leaves many stroke cases undiagnosed and many stroke patients untreated. Rapid initiation of thrombolytic and endovascular treatment is essential for best outcomes. Development of less expensive and more accessible diagnostic tools to quickly detect AIS has the potential to improve treatment times, thereby reducing the extent of brain damage, and significantly improving the quality of life for stroke survivors and their carers.

There have been massive advances made in the treatment of AIS in the acute neurointerventional setting in recent years, and mechanical thrombectomy has fast become a gold-standard of care for AIS. The recent flurry of activity in cerebrovascular medical device companies has resulted in important advances in thrombectomy device design in a very short time-frame. Future developments will be informed by further advancements in understanding the clots that cause ischemic stroke. Better understanding of the characteristics and composition of AIS clots is crucial to improved and optimized medical device design. There is marked heterogeneity in AIS clots, evident at gross inspection in terms of color, size as well as in histological composition and mechanical properties. Ongoing developments in stentrievers, aspiration catheters and other novel device design will continue to improve thrombus–device interaction to remove even the most difficult to extract clots, improving recanalization rates and patient outcomes. Clot characteristics are influenced by factors such as etiology (122, 123), and thrombolytic treatment (124). Further interrogation of AIS clots will help to advance the understanding of the pathophysiology of stroke, perhaps identifying novel leads for the development of new safer thrombolytic therapies.

Furthermore, there remains a huge need for other neuroprotective approaches to protect brain cells at risk due to acute trauma. Extensive previous studies have assessed the effectiveness of potential neuroprotective agents in animal models of AIS, but to-date none have shown real promise in human clinical trials. There have been many identified issues in the design of pre-clinical and clinical studies that may have contributed to the failure of neuro-cytoprotective agents in trials to-date, such as for example insufficient study size and the inclusion of patients who were unlikely to benefit (125). Excellence in study design is critical to ensuring future success. The availability of thrombolytic and endovascular reperfusion therapy has led to clear improvements in outcomes for those patients with quick access to a stroke center with EVT capability and who meet the criteria for treatment. However, more widespread availability of stroke centers with EVT capability is urgently needed worldwide, particularly in poorer countries and in sparsely populated regions. Furthermore, future clinical trials should assess the combined effect of neuro-cytoprotective treatments with thrombolysis and EVT, and the potential of multiphase adjuvant neuroprotective strategies (126, 127) in addition to sole administration. Despite intensive research, an approved neuro-cytoprotective drug is still far from the clinical reality, but the search must continue.

## Author Contributions

SP and KD contributed to the concept and design of manuscript. All authors contributed in drafting and reviewing of manuscript.

## Funding

This work was supported by the European Regional Development Fund and Science Foundation Ireland Grant Number (13/RC/2073_2).

## Conflict of Interest

The authors declare that the research was conducted in the absence of any commercial or financial relationships that could be construed as a potential conflict of interest.

## Publisher's Note

All claims expressed in this article are solely those of the authors and do not necessarily represent those of their affiliated organizations, or those of the publisher, the editors and the reviewers. Any product that may be evaluated in this article, or claim that may be made by its manufacturer, is not guaranteed or endorsed by the publisher.
